# Prediction of Insulin Resistance in Nondiabetic Population Using LightGBM and Cohort Validation of Its Clinical Value: Cross-Sectional and Retrospective Cohort Study

**DOI:** 10.2196/72238

**Published:** 2025-06-13

**Authors:** Ting Peng, Rujia Miao, Hao Xiong, Yanhui Lin, Duzhen Fan, Jiayi Ren, Jiangang Wang, Yuan Li, Jianwen Chen

**Affiliations:** 1 Health Management Center, Third Xiangya Hospital, Changsha, China; 2 School of Mathematics and Statistics, Hunan University of Technology and Business, 569 Yuelu District, Changsha, 410205, China, 86 18692269664, 86 88618571

**Keywords:** insulin resistance, machine learning, Light Gradient Boosting Machine, diabetes, diabetes prevention

## Abstract

**Background:**

Insulin resistance (IR), a precursor to type 2 diabetes and a major risk factor for various chronic diseases, is becoming increasingly prevalent in China due to population aging and unhealthy lifestyles. Current methods like the gold-standard hyperinsulinemic-euglycemic clamp has limitations in practical application. The development of more convenient and efficient methods to predict and manage IR in nondiabetic populations will have prevention and control value.

**Objective:**

This study aimed to develop and validate a machine learning prediction model for IR in a nondiabetic population, using low-cost diagnostic indicators and questionnaire surveys.

**Methods:**

A cross-sectional study was conducted for model development, and a retrospective cohort study was used for validation. Data from 17,287 adults with normal fasting blood glucose who underwent physical exams and completed surveys at the Health Management Center of Xiangya Third Hospital, Central South University, from January 2018 to August 2022, were analyzed. IR was assessed using the Homeostasis Model Assessment (HOMA-IR) method. The dataset was split into 80% (13,128/16,411) training and 20% (32,83/16,411) testing. A total of 5 machine learning algorithms, namely random forest, Light Gradient Boosting Machine (LightGBM), Extreme Gradient Boosting, Gradient Boosting Machine, and CatBoost were used. Model optimization included resampling, feature selection, and hyperparameter tuning. Performance was evaluated using *F*_1_-score, accuracy, sensitivity, specificity, area under the curve (AUC), and Kappa value. Shapley Additive Explanations analysis was used to assess feature importance. For clinical implication investigation, a different retrospective cohort of 20,369 nondiabetic participants (from the Xiangya Third Hospital database between January 2017 and January 2019) was used for time-to-event analysis with Kaplan-Meier survival curves.

**Results:**

Data from 16,411 nondiabetic individuals were analyzed. We randomly selected 13,128 participants for the training group, and 3283 participants for the validation group. The final model included 34 lifestyle-related questionnaire features and 17 biochemical markers. In the validation group, their AUC were all greater than 0.90. In the test group, all AUC were also greater than 0.80. The LightGBM model showed the best IR prediction performance with an accuracy of 0.7542, sensitivity of 0.6639, specificity of 0.7642, *F*_1_-score of 0.6748, Kappa value of 0.3741, and AUC of 0.8456. Top 10 features included BMI, fasting blood glucose, high-density lipoprotein cholesterol, triglycerides, creatinine, alanine aminotransferase, sex, total bilirubin, age, and albumin/globulin ratio. In the validation queue, all participants were separated into the high-risk IR group and the low-risk IR group according to the LightGBM algorithm. Out of 5101 high-risk IR participants, 235 (4.6%) developed diabetes, while 137 (0.9%) of 15,268 low-risk IR participants did. This resulted in a hazard ratio of 5.1, indicating a significantly higher risk for the high-risk IR group.

**Conclusion:**

By leveraging low-cost laboratory indicators and questionnaire data, the LightGBM model effectively predicts IR status in nondiabetic individuals, aiding in large-scale IR screening and diabetes prevention, and it may potentially become an efficient and practical tool for insulin sensitivity assessment in these settings.

## Introduction

Insulin resistance (IR) refers to the reduced sensitivity or responsiveness of target organs such as the liver, skeletal muscles, or adipose tissue to insulin. In order to maintain normal blood glucose levels, the body compensates by secreting increased amounts of insulin [1]. As a condition that can exist before the onset of type 2 diabetes, IR is not only one of the key mechanisms underlying the development of diabetes but also a major risk factor for various diseases [2]. According to the International Diabetes Federation, it is estimated that by 2045, there will be 783.2 million people affected globally, with the vast majority having type 2 diabetes [3]. Furthermore, IR is even more common. Data from the National Health and Nutrition Examination Survey in the United States show that approximately 40% of adults aged 18‐44 years have IR [4]. A study in China revealed that the standardized prevalence of IR in adults aged 25 years or older is 29.22% [5]. By scientifically assessing and managing IR, it is theoretically possible to effectively control the onset of diabetes. The first step is to identify IR. The high insulin clamp with normal blood glucose levels is considered the gold standard for assessing insulin sensitivity [6]. However, in practice, this method is time-consuming, expensive, and difficult to apply widely. The Homeostasis Model Assessment of Insulin Resistance (HOMA-IR; fasting glucose [mmol/L]×fasting insulin [μU/ml]/22.5) is regarded as an acceptable method for evaluating IR [7]. However, fasting insulin, one of its components, is not a routine test, making it difficult to obtain in community and grassroots settings. Based on laboratory indicators and lifestyle characteristics that are correlated with the occurrence of IR and are easily accessible, it is crucial to explore more convenient ways to predict the previous probability of IR in individuals. This approach could guide more precise IR screening and has the potential for prevention and control within the large population of China.

Machine learning (ML), with its data-driven methods, has already shown good predictive value in health issues such as arteriosclerosis [8], fatty liver [9], diabetes [10], and hypertension [11] . Obesity, dyslipidemia, lack of exercise, and sedentary lifestyles are considered risk factors for metabolic homeostasis [12]. By applying daily habits, basic information, and simple blood tests as features, we can build machine learning models in health check-up populations that are representative of the general population. The model of the ensemble learning algorithms based on decision trees, such as random forest (RF), Light Gradient Boosting Machine (LightGBM), Extreme Gradient Boosting (XGBoost), Gradient Boosting Machine (GBDT), and CatBoost, using multiple decision trees for predictions. They are capable of capturing complex nonlinear relationships, handling high-dimensional data, performing automatic feature selection, and mitigating the risk of overfitting through various mechanisms. In addition, these models offer strong scalability and are suitable for large-scale datasets, with most models having built-in overfitting prevention capabilities. These models can effectively predict IR and identify key predictive factors that are crucial for individual intervention. In addition, a retrospective cohort study can be used to determine the accuracy and practical value of these model results in distinguishing the onset of diabetes.

## Methods

### Study Population and Data Sources

The data used in this study were sourced from the electronic medical records of the Health Management Center at Xiangya Third Hospital, Central South University. The dataset includes participants who underwent physical examinations and questionnaire surveys at the Health Management Center between January 2018 and August 2022. A total of 17,287 adults with normal fasting blood glucose (FBG) were included, while individuals with FBG ≥6.1mmol/L or those with a history of abnormal glucose tolerance or diabetes were excluded.

The subsequent retrospective cohort consisted of individuals who completed their first health check-up at the Health Management Center of Xiangya Third Hospital between January 2017 and January 2019. These participants were followed for 5 years, with those diagnosed with diabetes at external hospitals or those who had completed ≥2 times FBG tests at the hospital during the follow-up period being included. Participants were required to be aged 18 years or older and have FBG <6.1mmol/L at the time of their first check-up. They also had to complete baseline assessments and surveys. Exclusion criteria included a history of abnormal glucose tolerance, diabetes, or gestational diabetes, as well as a history of tumors, pregnancy, or autoimmune diseases; and those who had used glucocorticoids or other relevant medications within the past month.

Using an IR prediction model, individuals in the cohort were classified as low-risk or high-risk based on their baseline data. Changes in FBG were then monitored. A FBG ≥7 mmol/L within 5 years or a diagnosis of diabetes at an external hospital was considered a positive result. A negative result was defined as maintaining normal FBG levels without a diabetes diagnosis at an external hospital over the 5-year follow-up period. Furthermore, by matching the ID card numbers, we excluded the participants who repeatedly appeared in the training set and the test set. Ensure this cohort was the different from the dataset used for model development.

### Characteristics and Definitions

The dataset includes a total of 102 features, which encompass physical examination measurements such as age, height, weight, BMI, waist circumference (WC), systolic blood pressure (SBP), and diastolic blood pressure (DBP). Blood test indicators include FBG, liver function markers (alanine aminotransferase [ALT], aspartate aminotransferase [AST], total bilirubin [TBIL], direct bilirubin [DBIL], serum albumin [ALB], serum globulin [GLO], albumin/globulin ratio [A/G], total proteintotal [TP], bile acids [TBA]), kidney function markers (serum creatinine [Sr], blood urea nitrogen [BUN], uric acid [UA], and lipid profile markers (total cholesterol [TC], triglycerides (TG], high-density lipoprotein cholesterol [HDL-C], low-density lipoprotein cholesterol [LDL-C]. In addition, information from questionnaires was collected, including past disease diagnoses, lifestyle factors (dietary preferences, smoking status, alcohol consumption, exercise habits, and work status), psychological status, sleep patterns, and health literacy related to basic medical knowledge. Insulin sensitivity was assessed using HOMA-IR=fasting glucose (mmol/L)×fasting insulin (μU/ml) / 22.5. A cut-off value of 2.69 was used, with a value ≥2.69 considered indicative of IR status [5].

### Data Processing and Statistical Analysis

We excluded features with more than 30% missing data. For features with less than 30% missing data, continuous variables were imputed using the mean, while categorical variables were imputed using the mode. Ultimately, 90 features and 16,411 samples were retained. The entire dataset was randomly split into a training set (13,128/16411, 80% samples) and a test set (3283/16411, 20% samples), with 2782 samples categorized as IR.

For statistical analysis, continuous data that conform to a normal distribution were reported as means (SDs), otherwise, were reported as quartiles. Chi-square tests were conducted on categorical variables to assess the significant association between categorical features and the dependent variable. *P*<.05 was considered statistically significant.

### Feature Engineering

#### Resampling

In this study, 3 resampling techniques, namely random undersampling, synthetic minority oversampling technique (SMOTE) oversampling, and SMOTE-Tomek combined sampling were experimentally compared during the dataset preprocessing phase. It was found that random undersampling performed best in improving the prediction accuracy for the minority class. Therefore, random undersampling was applied to the training set, where a portion of the majority class samples were randomly removed to reduce the majority class sample size. This approach helped mitigate model bias toward the majority class and improved overall classification performance.

#### Feature Processing and Algorithm Selection

To enhance model accuracy and efficiency, reduce computational costs, and avoid overfitting, feature selection was conducted. Before feature selection, Pearson correlation analysis was conducted for continuous features, while Kendall correlation analysis was used for discrete features. By examining the correlation matrix of the features, pairs of highly correlated features (with correlation coefficients >0.6) were identified, and perform dimensionality reduction on these highly relevant features. A total of 5 machine learning algorithms, including RF, LightGBM, XGBoost, GBDT, and CatBoost were used to construct predictive models on the training dataset. The model demonstrating the best performance across various evaluation metrics was selected from the baseline models (CatBoost was chosen based on experimental results). The CatBoost model was then trained, and the importance scores for each feature were determined. Features were added to the model in descending order of importance until the AUC score stabilized at its highest value with a specific number of features. At this point, no further features were added, and the model was constructed using the selected features.

### Parameter Optimization and Model Evaluation

In addition to addressing class imbalance through resampling techniques, the model parameters were adjusted to assign different weights to each class, thereby balancing the class distribution. For the RF, GBDT, and CatBoost models, the parameter class_weight = “balanced” was set, while for the LightGBM and XGBoost models, scale_pos_weight = “ratio of majority and minority class” was used. Bayesian optimization was used for parameter tuning. Bayesian optimization is a global optimization algorithm based on Bayesian inference, which iteratively updates the posterior distribution of the parameters to identify the optimal configuration and optimize the objective function. To evaluate the optimization effects and improve the model’s generalization ability, 10-fold cross-validation was performed. The experiments were repeated 10 times, and metrics such as area under the receiver operating characteristic curve were averaged to assess model performance. In addition, model performance was evaluated using the *F*_1_-score, accuracy, sensitivity, and specificity, while the receiver operating characteristic (ROC) curve and the area under the curve (AUC) were used to assess the model’s discriminatory ability. The Kappa value was also computed to evaluate the predictive capability of the model.

This research used several Python libraries and frameworks for data processing, feature engineering, model building, and hyperparameter tuning. The main tools used in the study are Python (version 3.6.4), NumPy (version 1.18.5), Pandas (version 1.1.5), SciPy (version 1.5.2), scikit-learn (version 0.24.2), LightGBM (version 4.3.0) XGBoost (version 1.5.2), CatBoost (version 1.1.1), and bayes_opt (version 1.4.0).

### Feature Importance Ranking

Shapley Additive Explanations (SHAP) values are a method used to interpret machine learning model predictions. SHAP assigns a value to each feature, quantifying its contribution to the model’s output. Positive SHAP values indicate that the feature has a beneficial impact on the prediction, while negative SHAP values suggest a detrimental effect. The absolute magnitude of a SHAP value reflects the extent of the feature’s influence on the model’s decision. In this study, we used SHAP (version 0.45.0) for explainable machine learning to enhance interpretability.

### Retrospective Cohort Validation of Model Application

Retrospective cohort data from a nondiabetic population were used as input to the aforementioned algorithms. The model outputs predicted the IR values for each participant, where a value of 1 was classified as high-risk IR and 0 as low-risk IR. Kaplan-Meier survival curves were used to conduct time-to-event analysis, comparing the 5-year diabetes incidence between the high-risk and low-risk IR groups. This approach further validated the model’s ability to accurately distinguish the onset of diabetes based on predicted IR levels.

### Ethical Considerations

This study was conducted in accordance with the Declaration of Helsinki and approved by the Ethics Committee of Xiangya Third Hospital, Central South University (approval no. 22206). All participants provided informed consent. The data were deidentified to ensure privacy protection.

## Results

### Characteristics of the Study Population

**Figure 1.**
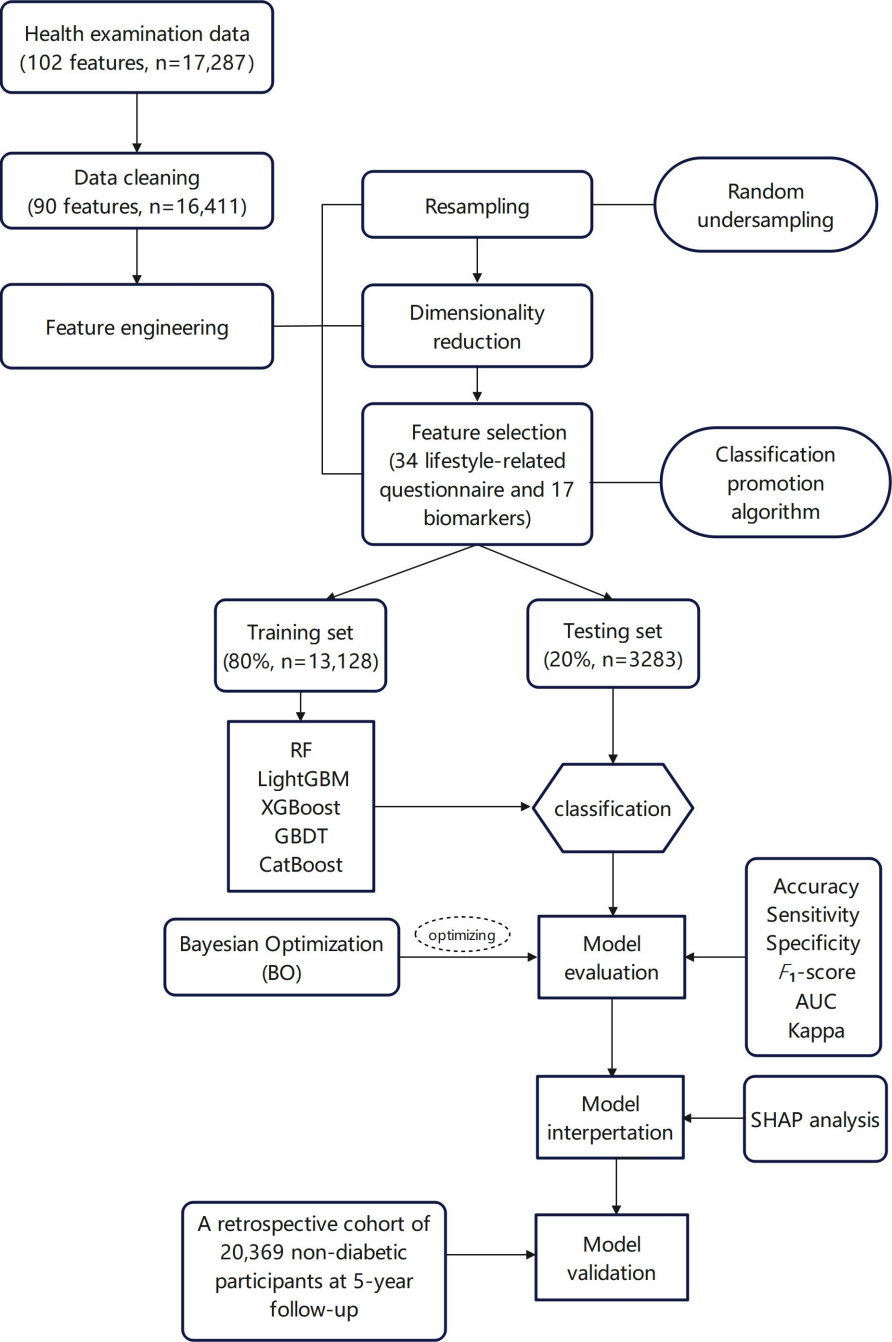
Flow diagram of the study. The flow diagram of the study shows the entire research process, from data collection, preprocessing, model training and testing to final model evaluation and validation. AUC: area under the curve; GBDT: Gradient Boosting Machine; LightGBM: Light Gradient Boosting Machine; RF: random forest; SHAP: Shapley Additive Explanations; XGBoost: Extreme Gradient Boosting.

The study flow chart is presented in Figure 1. A total of 16,411 nondiabetic individuals who underwent health screenings were included in this study, with an average age of 42.74 (SD 11.36) years. The sample comprised 8205 males and 8206 females. Overall, 17% of participants were diagnosed with IR, with a higher prevalence observed in males compared with females. In the IR group, levels of weight, WC, BMI, SBP, UA, ALT, AST, TG, and TC were significantly elevated compared with the non-IR group. Conversely, the average HDL-C level was significantly lower in the IR group (*P*<.05). In addition, lifestyle factors such as diet, physical activity, and health literacy exhibited significant differences between the 2 groups (Tables 1 and 2).

**Table 1.** Continuous characteristics of participants, including age, height, weight, waist circumference, BMI, blood pressure, and blood biochemical indicators, and a comparison of the means and SDs between the insulin resistance and non–insulin resistance groups, along with the corresponding *P* values to evaluate the association of each characteristic with insulin resistance.

	All (n=16,411), mean (SD)	IR^a^ (HOMA^b^-IR <2.69; n=13,629), mean (SD)	Non-IR (HOMA-IR ≥2.5; n=2782), mean (SD)	*P* value
Age (years)	42.74 (11.36)	42.84 (11.37)	42.23 (11.30)	.01
Height (cm)	163.50 (8.00)	163.14 (7.93)	165.29 (8.21)	<.001
Weight (kg)	63.80 (11.70)	62.21 (11.10)	71.48 (11.59)	<.001
WC (cm)^c^	81.30 (10.40)	79.82 (9.81)	88.53 (9.67)	<.001
BMI (kg/m^2^)^d^	23.85 (3.37)	23.30 (3.07)	26.54 (3.48)	<.001
SBP (mmHg)^e^	120.39 (14.83)	119.49 (14.7)	124.75 (14.62)	<.001
DBP (mmHg)^f^	73.80 (10.80)	73.08 (10.71)	77.02 (11.04)	<.001
BUN (mmol/L)^g^	4.72 (1.22)	4.71 (1.23)	4.79 (1.19)	<.001
Sr (umol/L)^h^	70.31 (18.15)	69.99 (18.52)	71.85 (16.12)	<.001
UA (umol/L)^i^	339.53 (92.01)	331.13 (89.53)	380.70 (92.91)	<.001
TBA (umol/L)^j^	4.22 (4.80)	4.18 (4.82)	4.44 (4.69)	.01
ALT (u/L)^k^	26.46 (25.62)	23.30 (24.73)	36.09 (27.21)	<.001
AST (u/L)^l^	24.89 (18.88)	24.50 (20.07)	26.83 (11.17)	<.001
ALB (g/L)^m^	46.62 (2.91)	46.63 (2.91)	46.60 (2.93)	.56
GLO (g/L)^n^	27.09 (3.68)	26.93 (3.65)	27.88 (3.71)	<.001
A/G^o^	1.73 (0.24)	1.74 (0.24)	1.70 (0.25)	<.001
TP (g/L)^p^	73.72 (4.22)	73.56 (4.19)	74.47 (4.30)	<.001
DBIL (umol/L)^q^	3.78 (1.34)	3.83 (1.35)	3.54 (1.26)	<.001
TBIL (umol/L)^r^	13.02 (5.06)	13.21 (5.11)	12.05 (4.70)	<.001
FBG (mmol/L)^s^	5.24 (0.42)	5.20 (0.42)	5.45 (0.40)	<.001
TG (mmol/L)^t^	1.70 (1.53)	1.55 (1.36)	2.44 (2.01)	<.001
TC (mmol/L)^u^	5.01 (0.94)	4.98 (0.93)	5.16 (1.00)	<.001
HDL-C (mmol/L)^v^	1.33 (0.30)	1.36 (0.29)	1.17 (0.24)	<.001
LDL-C (mmol/L)^w^	2.89 (0.79)	2.88 (0.78)	2.93 (0.84)	<.001

a IR: Insulin resistance.

b HOMA: Homeostasis Model Assessment.

c WC: waist circumference.

d BMI: body mass index.

e SBP: systolic blood pressure.

f DBP: diastolic blood pressure.

g BUN: blood urea nitrogen.

h Sr: serum creatinine.

i UA: uric acid.

j TBA: total bile acids.

k ALT: alanine aminotransferase.

l AST: aspartate aminotransferase.

m ALB: serum albumin.

n GLO: serum globulin.

o A/G: albumin/globulin ratio.

p TP: total protein.

q DBIL: direct bilirubin.

r TBIL: total bilirubin.

s FBG: fasting blood glucose.

t TG: triglycerides.

u TC: total cholesterol.

v HDL-C: high-density lipoprotein cholesterol.

w LDL-C: low-density lipoprotein cholesterol.

**Table 2.** Questionnaire characteristics of participants, including dietary habits, exercise, lifestyle, psychological status, and health literacy, and an assessment of the association of each characteristic with insulin resistance using the chi-square test, listing the chi-square values and *P* values.

Type and feature	Chi-square (*df*) value	*P* value
Diet		
Dietary preference	137.86 (13)	<.001
Daily meat intake	123.43 (4)	<.001
Fruit consumption	29.81 (4)	<.001
Coffee consumption	21.22 (4)	<.001
Nighttime snacking	54.97 (2)	<.001
Social dining	90.56 (3)	<.001
Legume and soy product consumption	13.40 (4)	.01
Fish or seafood consumption	7.24 (4)	.12
Sugary drink consumption	106.32 (4)	<.001
Dietary taste	125.78 (2)	<.001
Fat meat consumption	88.97 (4)	<.001
Animal organ consumption	25.31 (3)	<.001
Staple food structure	33.95 (4)	<.001
Binge eating	73.63 (1)	<.001
Daily vegetable intake	23.77 (4)	<.001
Milk consumption	13.20 (4)	.01
Egg consumption	1.83 (3)	.61
Exercise		
Physical exercise	56.60 (2)	<.001
Weekly exercise frequency	59.24 (3)	<.001
Exercise duration per session	54.73 (3)	<.001
Exercise type	122.97 (31)	<.001
Years of consistent exercise	60.50 (4)	<.001
Lifestyle		
Alcohol consumption	29.25 (3)	<.001
Alcohol frequency per week	25.38 (3)	<.001
Years of alcohol use	36.00 (4)	<.001
Alcohol quantity per session	57.59 (4)	<.001
Type of alcohol	13.95 (6)	.03
Time since quitting drinking	6.65 (4)	.16
Work physical demands	95.25 (4)	<.001
Workdays per week	3.85 (3)	.28
Working hours per week	3.36 (4)	.50
Smoking habits	42.55 (3)	<.001
Cigarettes per day	38.48 (4)	<.001
Years of smoking	56.12 (4)	<.001
Exposure to Harmful Substances	47.16 (18)	<.001
Sedentary time outside work	36.27 (3)	<.001
Regular meals	9.12 (3)	.03
Sleep quality	1.01 (2)	.60
Reasons for sleep disturbance	22.03 (12)	.04
Sleep duration	3.52 (3)	.32
Main symptoms of sleep disturbance	15.448 (12)	.22
Psychology		
Depression	0.10 (2)	.95
Difficulty concentrating	8.45 (3)	.04
Increased anxiety	1.57 (2)	.46
Burnout	1.89 (2)	.39
Depressed	3.19 (3)	.36
Anxious	0.18 (2)	.91
Anxiety and restlessness	0.56 (2)	.76
Irritable	0.76 (2)	.68
Impatience	2.07 (2)	.36
Health literacy		
Normal WC^a^	25.37 (3)	<.001
Active medical knowledge acquisition	22.63 (2)	<.001
Self-Monitoring of blood pressure and heart rate	20.81 (2)	<.001
Normal BP^b^	7.82 (1)	.01
Normal BMI	6.71 (1)	.01
Normal FBG^c^	1.71 (1)	.19
Normal T^d^	1.02 (1)	.31
Normal HR^e^	1.07 (1)	.30
Normal TG^f^	0.72 (1)	.39
Normal TC^g^	2.66 (1)	.10
Normal Salt intake	78.68	<.001
History of hypertension	127.39 (2)	<.001
Family history medicine	791.80 (509)	<.001
Observe urination and defecation	13.10 (2)	<.001
Bask	13.10 (3)	<.001
Seat belt usage	7.09 (2)	.03
Carrying emergency medication	3.35 (2)	.19
Health check-up interval	12.46 (5)	.03
Personal history disease	1.25 (1)	.26
FH: DM^h^	4.00 (1)	.05
Sex	151.26 (1)	<.001

a WC: waist circumference

b BP: blood pressure

c FBG: fasting blood glucose

d T: temperature

e HR: heart rate

f TG: triglycerides

g TC: total cholesterol

h FH: DM: family history of diabetes.

### Feature Engineering

In the training set, random undersampling was applied to the majority class. Among the “physiological features,” 4 highly correlated features: “BMI,” “height,” “weight,” and “WC” were subjected to dimensionality reduction, with “BMI” retained. Similarly, for the 2 highly correlated features “SBP” and “DBP,” only “SBP” was retained.

**Figure 2.**
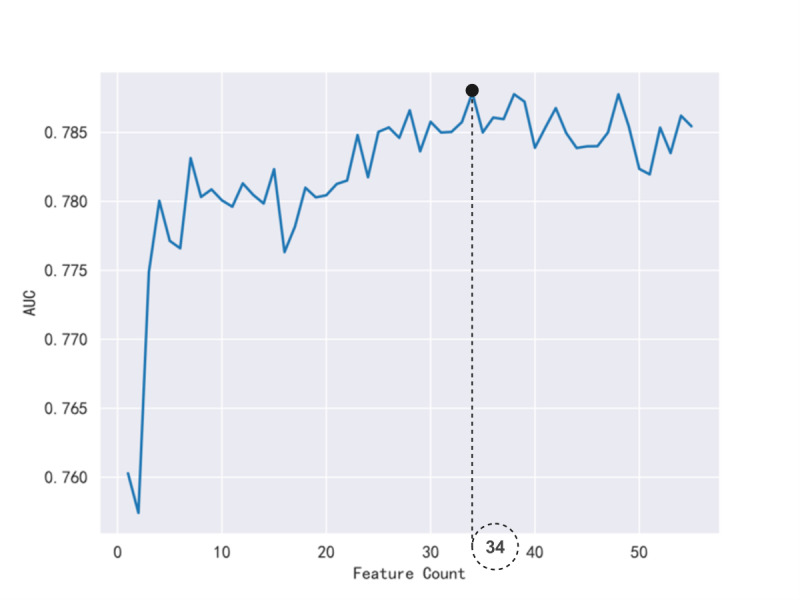
The model’s area under the curve (AUC) score on the test set. This figure shows how the AUC score on the test set changes with the number of features included in the model. After adding 34 features, the AUC score drops significantly, indicating that these additional features no longer contribute to model performance improvement.

Feature importance scores were computed for 55 lifestyle-related questionnaire features using the CatBoost algorithm. These features were ranked from high to low based on their importance scores and then sequentially input into the CatBoost model. After each addition, the model’s AUC score on the test set was recorded. As shown in Figure 2, the AUC score dropped significantly after adding 35 features, leading to the exclusion of 21 noncontributory questionnaire features. The 34 retained features, which showed significant contributions, include BMI, SBP, age, sex, family history medicine, exercise type, years of consistent exercise, exercise duration per session, weekly exercise frequency, dietary preferences, staple food structure, dietary taste, sleep duration, reasons for sleep disturbance, exposure to harmful substances, work physical demands, sedentary duration outside of work, consumption frequency of fruits, milk, meat, coffee, legume and soy products, sugary drink and fatty meats, daily vegetable intake, health check-up interval , self-monitoring of blood pressure and heart rate, observe urination and defecation, carrying emergency medications, regular meals, seat belt usage, bask, and awareness of normal salt intake and hazard ratio (HR).

In addition, the addition of 17 biochemical markers, BUN, Cr, UA, TC, TG, HDL-C, LDL-C, FBG, ALT, AST, TBIL, DBIL, TSP, ALB, GLO, A/G ratio, and TBA further improved the model’s accuracy, sensitivity, and specificity. Thus, the final feature selection for the model included the 34 lifestyle-related questionnaire features and 17 biochemical markers.

### Model Performance Evaluation and Feature Importance Ranking

**Figure 3.**
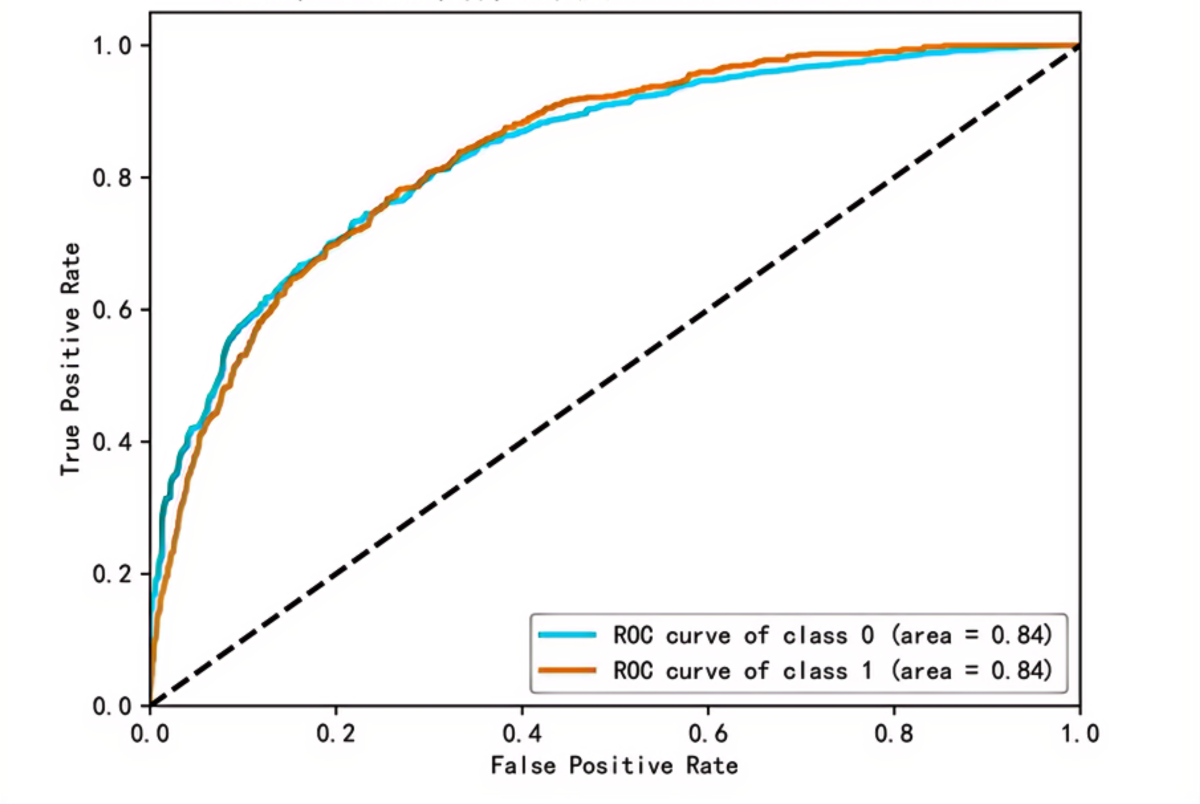
Prediction of insulin resistance in patients with insulin resistance by Light Gradient Boosting Machine, ROC in test set. The area under the curve is 0.8456, indicating that the model has good discriminant ability. ROC: receiver operating characteristic.

Based on the 51 selected features (34 questionnaire features and 17 biochemical markers), 5 ML algorithms were applied, RF, LightGBM, XGBoost, GBDT, and CatBoost to build and evaluate models using both the training and testing datasets. The performance metrics of the 5 ML models are summarized in Table 3. The LightGBM model demonstrated the best performance across all metrics, including accuracy (0.7542), sensitivity (0.6639), specificity (0.7642), *F*_1_-score (0.6748), and κ (0.3741). Although the AUC (0.8456) of the LightGBM model was slightly lower than that of CatBoost, LightGBM was determined to be the best model overall for predicting IR. The ROC curve for the best-performing LightGBM model is shown in Figure 3.

**Figure 4.**
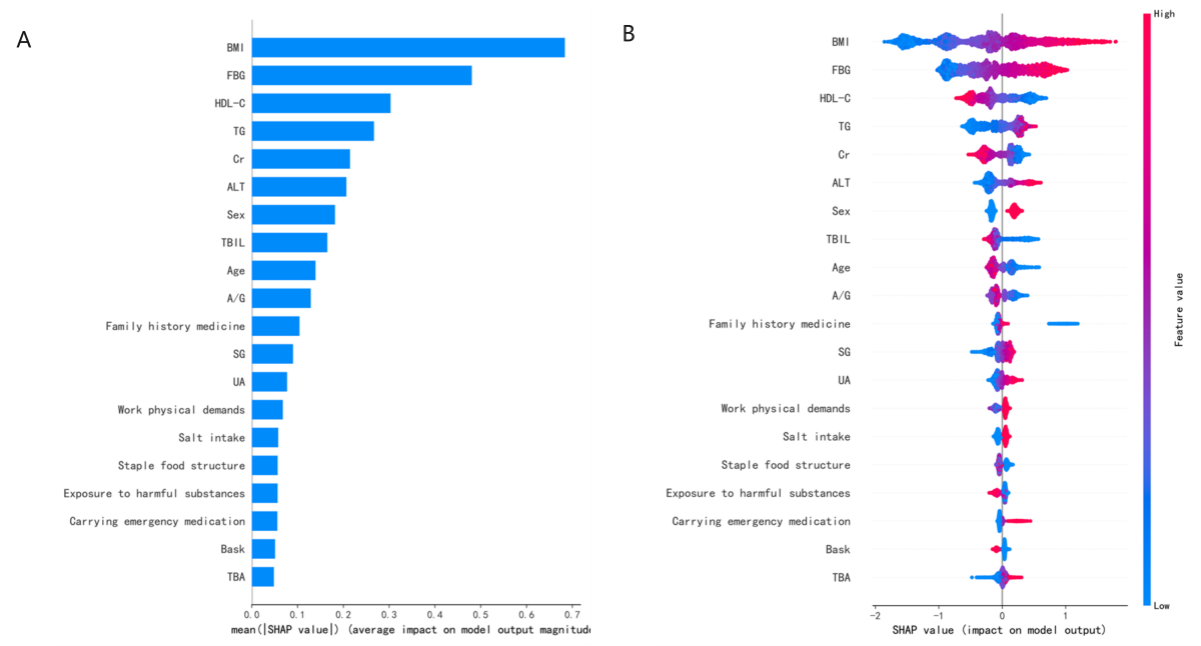
Detailed feature importance. (A) Feature importance by Light Gradient Boosting Machine. It shows the importance ranking of various features in the Light Gradient Boosting Machine model. The vertical coordinate (y-axis) shows the top 20 features and (B) shows the explanation of each feature impact on insulin resistance in the prediction model by the Shapley Additive Explanations (SHAP) values in the Light Gradient Boosting Machine algorithm. A/G: albumin/globulin ratio; ALT: alanine aminotransferase; Cr: creatinine; FBG: fasting blood glucose; HDL-C: high-density lipoprotein cholesterol; SG: serum glutamic; TBA: bile acids; TBIL: total bilirubin; TG: triglycerides; UA: uric acid.

SHAP values were used to rank feature importance in the LightGBM model, as shown in Figure 4A. The top 10 features influencing IR were: BMI, FBG, HDL-C, TG, Cr, ALT, sex, TBIL, age, and A/G ratio. Figure 4B provides a visual representation of the SHAP values for the top 10 features, where BMI, FBG, TG, ALT, and sex were positively correlated with IR, while HDL-C, Cr, TBIL, age, and A/G ratio were negatively correlated with IR.

**Table 3.** Performances of the 5 machine learning models using 51 selected features for participants.

Model	Accuracy_train	Accuracy_test	Sensitivity	Specificity	*F*_1_-score	AUC_train	AUC_test	κ value
RF^a^	0.8807	0.7344	0.6522	0.7516	0.6571	0.9552	0.8354	0.3451
LightGBM^b^	0.8503	0.7542	0.6639	0.7642	0.6748	0.9285	0.8456	0.3741
XGBoost^c^	0.9689	0.7460	0.6575	0.7556	0.6662	0.9966	0.8375	0.3588
GBDT^d^	0.9237	0.7505	0.6608	0.7598	0.6708	0.9799	0.8372	0.3668
CatBoost	0.8632	0.7493	0.6594	0.7576	0.6692	0.9445	0.8471	0.3637

a RF: random forest.

b LightGBM: Light Gradient Boosting Machine.

c XGBoost: Extreme Gradient Boosting.

d GBDT: Gradient Boosting Machine.

### Retrospective Cohort for Validation and Clinical Significance Assessment

**Figure 5.**
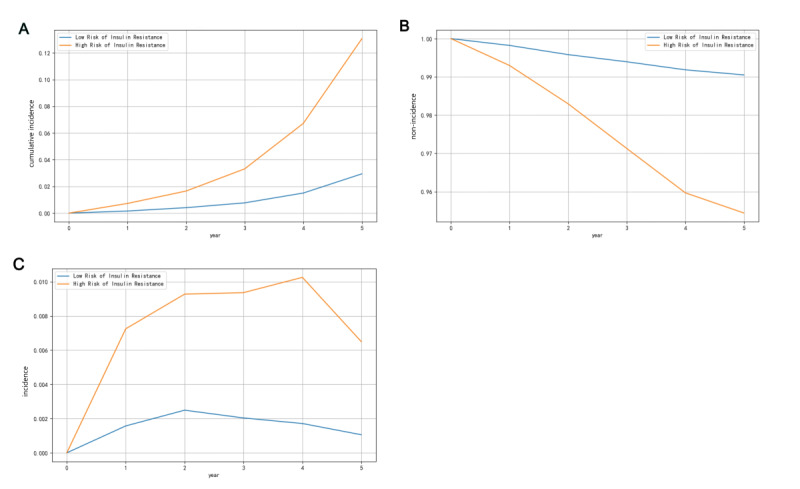
Clinical implication (incidence of diabetes) predicted by Light Gradient Boosting Machine algorithm from a retrospective cohort by the health management center. (A) Kaplan–Meier curve of cumulative incidence of diabetes in different states of insulin resistance. The high-risk insulin resistance group has a significantly higher cumulative incidence of diabetes than the low-risk insulin resistance group. (B) Kaplan–Meier curve of nonincidence of diabetes in different states of insulin resistance. The low-risk insulin resistance group has a significantly higher probability of not developing diabetes than the high-risk insulin resistance group. (C) Incidence of diabetes in different states of insulin resistance. The incidence in the high-risk insulin resistance group is significantly higher than that in the low-risk insulin resistance group, with a sharp increase after the second year.

The retrospective cohort dataset was used to validate the differential occurrence of diabetes in populations stratified by the model. A total of 20,369 nondiabetic participants were continuously monitored (5101 individuals classified as IR high risk by LightGBM and 15,268 individuals classified as IR low risk), with the incidence of diabetes compared from baseline to 5 years. Out of 5101 high-risk IR participants, 235 (4.6%) developed diabetes, while 137 (0.9%) of 15,268 low-risk IR participants did. This resulted in a HR of 5.1, indicating a significantly higher risk for the IR high risk group compared with the IR Low Risk group. Figure 5A clearly illustrates the relationship between the cumulative incidence of diabetes in the high- and low-risk insulin resistance groups. The high-risk group exhibited a significantly higher cumulative incidence of diabetes compared with the low-risk group, particularly after the second year of follow-up, when the rate of increase accelerated. This suggests that individuals in the high-risk group have a higher likelihood of developing diabetes, and the cumulative effect of this risk becomes more pronounced over time.

The curve for the low-risk group starts at 1 (indicating that 100% of individuals were free of diabetes) and gradually declines over time, although the decrease is relatively small. In contrast, the high-risk group shows a much more rapid decline, especially after the second year, with a more noticeable downward trend (Figure 5B). The line chart depicting the diabetes incidence rates across different IR risk groups (Figure 5C) shows that the growth rate of incidence in the high-risk group is significantly higher than in the low-risk group. The incidence in the high-risk group rises sharply after the second year, reaching its peak by the fifth year. These findings suggest that individuals with high insulin resistance are more likely to develop diabetes within a shorter time frame.

## Discussion

### Principal Findings

This study applied multiple machine learning methods using a health check-up database from southern China. Based on questionnaire data and easily accessible, low-cost laboratory measurements, a highly effective predictive model for IR was developed in a population with normal FBG. This ensures that the data required for model implementation is readily available, without the need for additional, expensive diagnostic procedures. This model holds significant value for precision screening of IR and for targeting interventions aimed at managing individual risk factors associated with IR. Compared with other models, LightGBM demonstrated a clear overall advantage in terms of its parameters. LightGBM is a gradient boosting framework that uses decision tree-based learning algorithms. It offers several benefits, including faster training efficiency, lower memory usage, and higher accuracy. In addition, it supports parallel learning and is capable of handling large-scale datasets [13].

### Comparison With Previous Work

The previous studies have developed insulin resistance prediction models targeting different ethnic groups and populations, which proves that researchers recognize the necessity of conducting IR prediction. Tsai integrated the National Health and Nutrition Examination Survey public database in the United States and the MAJOR database in Taiwan, China, and established the HOMA-IR ML (XGBoost) prediction model for adults in the two regions. The AUC reached 0.87, the important feature spectrum revealed by SHAP analysis was similar to our results, confirming the determinative roles of factors such as BMI, FBG,TG, HDL-C, and sex [14]. Before this, the team also developed an IR model using samples from 1,229 chronic kidney disease patients sourced from the National Health and Nutrition Examination Survey database. The model included various nutritional and micronutrient indicators, and the overall evaluation metric showed that the XGBoost model achieved an AUC of 0.78 [15]. Zhang et al [16] developed a LightGBM model for IR in a cohort of nearly 10,000 adults aged 40 years or older from certain communities in Hubei, China, with an ROC score of 0.794. The most significant features included FBG, BMI, WC, TG, and sex. In a South Korean study involving 8842 residents, in addition to measurement and laboratory indicators, high-cost, high-quality features such as genetic risk scores, food frequency questionnaires, and nutrients were included, the best-performing model, XGBoost, achieved an AUC of 0.86 [17]. However, these studies either use expensive features or fail to address multicollinearity issues between features. For example, physiological knowledge and typical data support a strong correlation between BMI and waist circumference [18], but these studies did not account for multicollinearity, which could lead to overfitting.

In our preliminary model, using only low-cost questionnaire features did not yield the expected results. However, when laboratory indicators of liver function and blood lipids were added, the performance improved significantly. Both feature importance rankings based on LightGBM and SHAP value calculations showed that BMI, FBG, HDL-C, and TG were among the top 4 most important features. In fact, a 2003 consensus from the American College of Clinical Endocrinology [19] formally defined IR syndrome as a multisystem disease centered around IR, with key elements including IR or hyperinsulinemia, with or without related cardiovascular-endocrine metabolic abnormalities. These elements include overweight BMI, abnormal glucose tolerance, hypertension, elevated TG, or reduced HDL-C, based on the established epidemiological correlation between IR and these indicators [20,21]. From a pathophysiological perspective, IR and these metabolic indicators have an explainable causal relationship. For example, adipokines secreted by adipose tissue, such as adiponectin [22], tumor necrosis factor alpha [23] can modulate insulin sensitivity through the insulin signaling pathway. In addition, hydrolysis products of elevated triglycerides, such as free fatty acids, can induce insulin resistance by inhibiting insulin signal transduction and reducing the number of insulin receptors on target cells [24]. Low HDL-C improves insulin resistance through reverse cholesterol transport and anti-inflammatory effects [25]. We also found that liver function indicators, such as ALT, bilirubin, and the A/G ratio, play a significant role in IR prediction, with fatty liver being closely linked to IR [26]. These 3 abnormal indicators are common in individuals with fatty liver disease [27], which may indirectly reflect IR. In addition, kidney function indicators showed correlations, which could be related to factors such as physical inactivity, chronic inflammation, oxidative stress, vitamin deficiencies, adipose factor imbalances, and changes in the gut microbiota in populations with kidney damage [28]. Finally, we applied the best LightGBM model results to baseline IR stratification in a retrospective cohort, further validating the significant impact of IR prediction on diabetes incidence. This aims to clarify the practical value of such models. By comparing the incidence of diabetes in low-risk and high-risk IR groups, we observed that individuals at high risk for IR were more likely to develop diabetes in a shorter period, confirming the model’s value in assessing blood glucose metabolism.

### Limitations and Future Directions

Despite the promising results in IR prediction, our study has several limitations. First, the data source is relatively limited in terms of geographical diversity. Our data came from a health check-up database from the Han Chinese population in Hunan Province in Central Southern China, which may impact the generalizability of the model to other regions or ethnic groups. Future studies could address this by incorporating data from different regions and ethnic populations to further validate the model’s generalizability. We would try to develop these ML models into user-friendly web pages or applications that are accessible to the general public and primary care providers, getting more input information and feedback to optimize our models, which is virtually a significant advantage of ML. Second, we tested the clinical implications of this trained model with a retrospective cohort, and have not yet explored the predictive efficiency of the model in prospective research, and we plan to perform it in a follow-up study in the near future. Third, the diagnosis of diabetes in the cohort was based only on the results of FBG, rather than oral glucose tolerance test or combined with postprandial blood glucose or glycated hemoglobin. However, we hope to base diagnoses on the latter as well in further research in the near future. Finally, although 5 ML algorithms were used in this study, there may be other algorithms with better performance that are currently available or soon to be developed. It would be necessary for us to further iterate models with more promising algorithms to improve the predictive performance of the models in the future.

### Conclusions

In conclusion, the ML models using the LightGBM algorithm are efficient in predicting IR status in nondiabetic individuals. By leveraging low-cost laboratory indicators and questionnaire data, the model can accurately assess the current IR status in individuals with normal blood glucose levels, helping identify those at high risk of progressing to diabetes within large-scale populations.

## Abbreviations

A/G: albumin/globulin ratio
ALB: serum albumin
ALT: alanine aminotransferase
AST: aspartate aminotransferase
AUC: area under the curve
BUN: blood urea nitrogen
DBIL: direct bilirubin
DBP: diastolic blood pressure
FBG: fasting blood glucose
GBDT: Gradient Boosting Machine
GLO: serum globulin
HDL-C: high-density lipoprotein cholesterol
HOMA: Homeostasis Model Assessment
IR: insulin resistance
LDL-C: low-density lipoprotein cholesterol
LightGBM: Light Gradient Boosting Machine
ML: machine learning
RF: random forest
ROC: receiver operating characteristic
SBP: systolic blood pressure
SHAP: Shapley Additive Explanations
SMOTE: synthetic minority over-sampling technique
Sr: serum creatinine
TBA: bile acids
TBIL: total bilirubin
TC: total cholesterol
TG: triglycerides
TP: total proteintotal
UA: uric acid
WC: waist circumference
XGBoost: Extreme Gradient Boosting
